# Hysteria

**Published:** 1854-06

**Authors:** Sanford B. Hunt


### SELECTIONS.

*Hysteria. Considered with reference to its mental phenomena.* By Sanford B. Hunt, M.D.

It is my purpose in this article to speak of the hysteria with reference to those mental phenomena which it involves, and which are perhaps the most important of the numerous symptoms of the disease. The occurrence of a hysterical convulsion, of greater or less severity, is, in itself, a matter of no great moment—our patient may be, on the day succeeding, fit for labor or society, with no impairment of physical or mental health.

But a succession of these attacks, either convulsive or comatose, if long continued, is apt to involve a train of mental phenomena of the most interesting character, though that interest is too often derived from the curiosity with which we watch the gradual decay of mental vigor, and the substitution for it of suspicion, jealousy of friends, or some other form of perverted reason.

And the consideration of hysteria has, of late years, become more necessary, from the fact that our abilities for appreciating its symptoms have recently been greatly enlarged, in the developments which have attended those moral epidemics which have lately swept over the civilized world.

The student of nervous pathology must often recognize in the insanities of Swedenborgianism, Homœpathy, Spiritualism, and especially Electro-Biology and Mesmerism, a train of mental *and of bodily* symptoms, to some extent identical with those of hysteria. To trace these resemblances is our task. In the pursuit of them we may overstep the boundaries of medical literature, and search in the moral movements of the day, or in the field of secular literature for facts germain to our investigation.

Among the symptoms to which I shall direct this attempt at explanation, and which every practitioner must occasionally have recognized. are—1, hysterical coma, or hypnotism; 2, somnambulism; 3, extreme credulity of statements unsupported by evidence or foregone experience; 4, ecstacy; and especially, 5, extraordinary monomania for the deception of those around them. The phenomena of these conditions are now sufficiently understood to render them applicable to our ideas of the necessities of treatment, and it is not too much to assume, that he who looks upon hysteria as a mere manifestation of derangement of the cerebrospinal system, dependent on organic causes, is not entirely competent to its proper management.

Convulsion is not the most common or constant of hysterical symptoms. The same is true of all those bodily symptoms we are accustomed to look upon as hysterical. Although some of them are usually present, yet no one of them seems essential to the disease—it has no one intrinsic or pathognomic symptom, unless, indeed, the absence of laryngeal spasm may be considered a negative symptom.

It is my belief that the mental symptoms of hysteria are as constant and as peculiar to the disease, as the physical. Setting aside the question of their relative frequency, they are in themselves far more important in their results. I have seen imbecility, attempts at suicide, suspicion of friends, desertion of families, in which hate was substituted for former love, absurd religious belief, and many other disastrous consequences resulting from this affection. All these things tend to confer importance on the subject of our essay.

The fact that medical literature contains so little upon the mental forms of hysteria, is owing principally to the circumstance that very few physicians have made the philosophy of mind a part of their studies. But this paucity of mental research is redeemed by the circumstance that sundry observers collaterally connected with medicine have written largely and well upon subjects which have so close a connection as to be almost identical. De Boismont on Hallucinations, Sir David Brewster's Natural Magic, Sir Walter Scott's Demonology, Abercrombie's Intellectual Philosophy, and last and greatest, the article in the Quarterly Review of October, 1853, attributed to Dr. Carpenter, have much matter that bears upon my inquiry. There are numerous other works with which I have only an acquaintance through critical notices, or occasional notices or extracts in other authors, but from which I have sometimes thus obtained at second hand some valuable facts or suggestions. These are, Braid's Experiments in Hypnotism, Falcot, Du Suicide et de l'Hypochondrie, Dendy's Philosophy of Mystery, Esquirol, and Forbes Winslow's Anatomy of Suicide. To these should be added a host of articles in medical, religious and spiritual journalism, and lastly an old book which adorns my shelves, and which has no equal on its peculiar subject—Sir Gilbert Blane's Medical Logic.

1. *Hysterical Coma.*—One of the most common manifestations of hysteria is this form of stupor. The patient lies as in a trance, their eyes may be either open or closed, their pupils usually responsive to the action of light, the muscular system either in a condition of paralytic repose, or else subjected to tonic spasm, sometimes amounting to opisthotonos, almost always to trismus. Clonio spasms are not usual in this form. Sometimes the rigidity is very permanent, and in cases where the patient maintains any position, however painful, in which she may be placed, the disorder is, somewhat fancifully, removed from the category of hysteria, and classed as catalepsy. I have never met with a well developed catalepsy, though I have sometimes induced a patient to simulate its symptoms very accurately.

In order to express my idea of the mental condition which obtains in hysterical coma, I would premise that a *feeble will* is the predisposing or constitutional cause of the disease. For a fuller illustration I quote from an article I communicated to this journal in December, 1852:

"These conditions then are but different stages of the same action. Irritation is the prodrome of congestion. Chill is a symptom of congestion, and spasm is but a magnified chill.

"In the child, the principal office of the brain is to govern the motive power. It has not yet attained to its highest intellectual function.

"A child rarely has chill—in fact those causes which would suffice to cause chill only in the adult, will give us convulsion in the infant. Children are more subject to cerebral, than to spinal diseases. The child has not learned to use its muscles—the will has no control over them—there is a lack of that proper balance of power between the brain and spinal cord, which alone can insure an equality of action. In the adult, chill takes the place of convulsion because the brain has acquired such power as enables it by efforts of the will to controll those spasmodic actions to which the child must submit.

"Women are much more liable to convulsion than men. Their wills are not so strong. Many funny things have been said of "women's will," but the strength of their will lies in its very weakness. It is a fact worthy of notice, that rotundity of outline, and symmetry of figure are the children of the spinal system; while activity and strength of the cerebral functions pare down the rotundity, and sharpen the outline of the figure. Witness the shriveled limbs and sharpened faces of big-headed and precocious children. It is uncommon to find great beauty of person and strength of intellect in the same individual. Ever since Narcissus, bending beside the fountain, became enamoured of himself and changed into a daffodil, great beauties and great fools have been synonymous."

The immense importance of the will in the government of mental actions is very clearly set forth in the article, the authorship of which is referred to Dr. Carpenter. It is the master-faculty, and drives the other four-in-hand. Inasmuch as any sensory impression is immediately referred to the perception, and requires an effort of the will in order to recollect other similar perceptions, that it may be classified and compared with experience; it is evident that the will being absent, or concentrated upon any one "dominant idea," the spinal system is left to its own unguided functions. We have then only reflex action and our patient is for the time in the condition of a decapitated frog.

Now in the ordinary hystery characterized by chronic spasm, without unconsciousness, we have merely partial defects of the will, which is so far withdrawn that the spinal reflex system responds by convulsive action to impressions made upon it by some irritated nerve, most commonly located in the uterine system.

But in hysterical coma we have, beside this absence of will, possession of the mind by a "dominant idea;" which has a strong family likeness to the devils of scripture, who "possessed" so many in those times of religious enthusiasm. This dominant idea most ordinarily consists in a fixed belief on the part of the patient that she is entirely unable to move her limbs, to speak, or to swallow. This is not an intentional deception, save in exceptional cases; but, as happen in Electro-Biology and Mesmerism, the mind of the patient is very much under the control of those around. If they, too, believe in the inability to move, the patient acquires new strength in her absurd conviction. Under such circumstances I have seen a lady whose only organic disease was a slight ulceration of the os uteri, remain for thirty hours without speech or motion.

The treatment of this form is simple. The leading fact of which we may take advantage, is the substitution of our own for her volition. Many will recollect the positive assurances which Biologists use in controlling their "subjects"—"you *cannot* raise that handkerchief from the table!" said one of these itinerant lecturers. And the subject could not, merely because his mind was possessed by the belief that he could not.

To get rid of this dominant idea, to substitute for it another and more truthful one, is the task of the physician. A very learned and intelligent physician of my acquaintance has the following very practical and sensible mode of treatment. Whatever he has to say he says loudly and distinctly, in the presence of his apparently insensible patient. He assures the friends that there is no cause for alarm, that she will regain her senses without any medication, that it is not necessary to watch by her, and that, in fact, she will, if left alone and undisturbed during the night, be found to be much better, and quite sensible in the morning. And his prediction is sure to be verified if he can induce the friends to follow his directions. But usually they are too much alarmed to consent to such a system of neglect. Our friend then assures them that he can restore consciousness immediately if they wish it. A tub of cold water is placed beside the bed. the patient's head and shoulders dragged over it, and liberally showered with water from a pitcher, dashed forcibly on her head, with occasional failures to hit the sinciput accurately, and a consequent deluge of the cold shower down her back, or into her bosom. This process is accompanied by loud and positive assertions that "she is coming out of it," that "ten minutes will bring her around right," and less than that time is always sufficient. The patient begins to feel "a little better," and the amendment is greeted by a more vigorous shower until at last the "dominant idea" is superseded by the very rational one that she is extremely cold and wet, and miserable. A return of the coma is prevented by the parting direction to shower her again if necessary. It is unnecessary to do more than to call to mind here that epidemic of hysteria in a German hospital, which was so suddenly checked by the instructions of the physician, *given in the presence of the patients*, to apply the actual cautery to the first person who showed convulsive symptoms. The red-hot iron became the "dominant idea."

In a case, occurring some years ago, where the coma and rigidity were so profound and immovable that I was inclined to consider it a dangerous cerebral disease, my anxiety was very much relieved by the involuntary, but ludicrously disgusted face, which attended a charitable attempt I made to spoon in a very nauseous cathartic through a hole left by an absent tooth. The shudder which ran over her was undoubtedly an unfeigned convulsion, and convinced me that she was at least sensible to impressions on the gustatory nerve. Yet this same patient had been bled and cupped without wincing. The clue afforded me by the occurrence narrated, led me to examine the rigidity; which I proved to exist only when she expected some one to touch her. She soon recovered under a different management; though she was liable to returns of the malady whenever any family jar occurred.

Now this condition was one of self-deception, and differs very little from the biological or mesmeric condition It can be produced artificially by the familiar means used by lecturers. It is a conceded physiological fact that if the eyes are directed upwards at some bright object, placed so near as to converge the axes of the eyes so much as to make them painful, a condition supervenes, the main characteristic of which is an abolition of the will, and a subjection of the subject's beliefs and actions to the violition of those around. Mr. Braid, a surgeon of Manchester, England, has experimented largely upon this curious condition, and has adadd to it the legitimate means of medical treatment. The "passes" used by mesmerists are merely means of increasing the soothing monotony of the ocular direction, or, secondarily, of fixing the attention of the "hypnotized" patient or any particular point, or organ. Thus, in a lady suffering from drying up of the lacteal secretion, Mr. Braid, by a few "passes" over the breast, restored the gland to a full and overcharged secretion of milk. This is not strange when we recollect how much the urinary, lachrymal, salivary, spermatic and other secretion are under the control of fixed attention, or dominant ideas. We may add that the Edinburgh Monthly Journal of Medical Sciences testifies to Mr. Braid's scientific character and credibility.

2. *Somnambulism.*—If we confine ourself to the narrow definition of this word included in the phrase "sleep-walking," we have no such apmptom in hysteria. But the condition is so analogous as to be worthy of notice in this connection. During sleep the will is dormant. The trains of ideas which make up our dreams are derived from suggestions, over which the will has no control. The action of the mind is "automatic," each thought suggesting its succeeding thought. In this condition a dominant idea has power to govern the actions of the sleeper. Destitute of judgment to connect, or compare his experiences, he acts upon the suggestions afforded him. In this, as in all cases where the body acts automatically, it has an increased precision of muscular movement, which enables him to accomplish acts which would be impossible to the unaided senses.

I have seen a case of epileptiform hysteria, (reported in this Journal in Dec., 1851, case 10,) in which the *petit mal*, or lesser form of epilepsy, seemed to be continued into a somnambulic condition. She would on these occasions, walk out of the house, like one in a trance, her eyes turned upward, and sometimes go a distance down the street, or into neighboring houses. During these trances she talked freely and sensibly, except at the moment of recovery, when she would become confused, and much distressed and surprised to find that she had made herself thus prominent. The condition was, in every respect, similar to somnambulism, and was but slightly different (if at all) from the mesmeric state. This patient had been subject to convulsions, called epileptic, and supposed to be incurable, from childhood. They were frequent and violent. Whatever may have been the early cause of the disease, it was finally removed, apparently, by blistering her spine, which was somewhat tender. A local uterine difficulty had been previously remedied by appropriate means. More than three years have elapsed since I saw my patient, but I learn that her health continues good, and that she has been free from any convulsive seizures, with one or two exceptions two years since, attributable to mental anxiety.

Mental causes had much to do with this case. She had an extravagant faith in my ability to relieve her, (a fact which may be accounted for by the fact that she was then rapidly approaching to imbecility !) and I did not hesitate to promise very largely.

3. *Extreme Credulity.*—This is a common symptom of this disorder. It is one which may be said to constitute both the bane and antidote of hysterical treatment. Thus, it is the foolish belief in an inability to move the limbs which is the principal feature of those bed-ridden hypochondriacs, who are the best of physicians. It does not always or generally amount to complete inability to move, but to a feeling of incapacity for labor, or exertion. Every person has this more or less; all feel their "Incompetent days." All physicians must have noticed how strong an influence the mental constitution, or in better words, the force of the will has upon that form of congestion called spinal irritation.

Some patients will endure, without abandonment of their daily avocations, a degree of spinal irritation absolutely wonderful while another, with a really trivial form of the malady, succumbs entirely to the burdensome feeling of malaise; and wanders about from doctor to docter, a very monomaniac for physic. That the condition of the mind is here the real point of attack for remedial measures, I hope to make evident my brief narrations of a few out of many cases.

Mr. D , a formerly robust and hard-laboring farmer, had been for two years unable to leave his house; and for much of that time under the care of physicians, and confined to his bed. He was peevish and irritable, but easily managed by his wife, who had complete control over him * His symptoms were merely general complaints of debility. I could find no disease of any organ, and as he had previously been under every conceivable form of treatment, I told him I did not know what to do, and would therefore do nothing at all.

Some months had elapsed, when I was called to him in the night to check a profuse epistaxis caused by sitting over the fire all day. Finding that the hæmorrhage was not alarming, I declined to do anything to stop it, assigning reasons without number for my course; the principal of which was, that this was a "critical hæmorrhage," indicating an effort of nature to establish health. The nose bled moderately through the night, my patient looking upon each drop as a substraction of so much disease from

*It is remarkable that in nearly, perhaps all, cases of mental derangement, from the slight form now under consideration to pronounced and violent insanity, some one person has a controlling influence over the unhappy mind of the patient.

his system. The next day I prescribed for him the Tinct. Ferri Muriatis, a positive assurance *that it would cure him*, and I even refused to admit a doubt as to its success. The result was very happy. He journeyed to his hog-pen in a day or two, then to his barn, and soon re-assumed the management of his farm, with its daily labors.

I have known a family of four brothers; all of them men of strong muscular development, accompanied by a pliable and easily influenced moral character; who have none of them escaped from this form of hypochondria. One of them was cured after seven years of idleness by a fit of passion, which seemed to arouse his sleeping will; two others recovered by moral means; though one of them is still subject to periods of melancholy and depression, while the fourth has for some dozen or fifteen years maintained a comfortable illness, which he encourages by a large use of advertised nostrums.

Although we must confine the term hysteria, etymologically, to the female, it is often easy to recognise its principal characteristics in the male. The slightest local uterine disease which so often occasions convulsion and other nervous symptoms in the woman, has its counterpart in the infantile convulsion incident to teething, or irritant ingesta. Stokes mentions convulsions in the adult arising from gastric irritation, and I have known the most frightful convulsions to occur in an adult man, from the presence of irritating or indigestible food in the intestines. I cannot be mistaken in this, for the fits occurred with very many repetitions, in which I searched carefully for the cause, and which I unfailingly cut short by cathartics, or enemata. The crude ingesta were often discoverable in the stools.

It was from this case reported at length in this Journal for December, 1852, that I was led first to recognize fully the influence of the will over disturbed nervous action. I called this case epilepsy, because there was some laryngeal spasm, but, aside from that feature, it did not differ from hysteria. I will quote a brief passage, illustrative of the comparative strength of the will and physical forces.

"The case had now become pronounced. The small aberrations from muscular control which we have called *petit mal*, have magnified themselves into the *grand mal*. He has epilepsy from eccentric causes, and in a form of unusual severity. He knows no relief from pain so severe as to cause the most terrific outcry, save when he looses sensation in a convulsion equally terrible. It is no unusual thing for crude food to cause cramp, but why should this cramp extend to the voluntary muscles, until, gathering strength, it sweeps down all the barriers of mental control and consciousness? The answer is to be found in the fact of his strong spinal system and weak brain An intellectual man of more feeble frame, would bear this pain without yielding up to any great extent his control over the voluntary muscles. This statement will find confirmation in the further history of the case.

"*Aug.* 31*st.* Vomited during the day quantities of undigested fruit with some relief. Movements of the bowels occurred and he was more easy though having occasional returns of convulsion till

"*Sept.* 1*st.* All the symptoms returned with increased violence. There was, in the character of the fits, some variations from true epilepsy. The *aura* was distinct in the shape of pain in the bowels, and sometimes prolonged for many minutes. Sometimes during the *aura* I sat by him exhort ng him to fortitude under his sufferings, *and to efforts of the will to keep of the fits.* Sometimes *by bringing all his resolution to bear, he was able to stave off the paroxysm*; but as the pain increased in intensity, he gave way, and found relief in the lethe of unconsciousness. For four or five days, at least one-half of his time was spent in convulsion, the other half in pain. Generally, after the fit there was heavy inspiration, and then delirium, during which he recollected the events of his fit, but saw them in a distorted light, manifesting the greatest antipathy to those who had held him on the bed, and much affright when those persons came into the room—the fright being sometimes sufficient to cause renewed convulsion. Afterward there was during the paroxysm a kind of of semiconsciousness, like that observed in hysterica—and this reminds me that his urine (which was removed entirely by the catheter) was very copious and limpid."

A brief mention of another case will suffice to complete the illustration of credulity as a feature of hysteria. In March, 1850, I was called to treat a Mrs. S., who for three years had been confined to her room or bed. I found that she had been intelligently treated by the physician who had preceded me for ulcerative abrasion of the os uteri. I found, also, that under that treatment she had very much improved as to local symptoms, but did not gain strength. Her local symptoms were again present, though with decreased severity. Assuming to make her case one of unusual study, I declined expressing any opinion as to the probability of her recovery, or of my ability to benefit her. After a few days of this non-committalism, I confidently assured her that her recovery was a matter of certainty. She did recover under a treatment directed entirely to the local disease, and so far as my information extended, differing in no essential particular from the management which had previously proved a failure.

It is the custom to ascribe these cases to Hope, but it is only through the re-invigoration of the will that hope can reach disease. The reputation attained by many water-cure establishments is owing to the liberal use of promises of cure, to the begetting of a faith, which, by rousing a feeble will, secures its own fulfilment. I am the more convinced of this, from the fact that I have known several cases of uterine disease to return from these places very much benefitted to bodily health. and able for the first time in years, in some instances, to perform their household duties; while the uterine disease remained *in statu quo;* unreached, and uncured; but happily existing almost unnoticed by the patient.

Like her who had for many years an issue of blood, and touched the hem of the Saviour's garment, their faith had made them whole.

4. *The Condition of Ecstacy*—Will be but briefly noticed as having but a narrow bearing on the treatment of disease. It generally exists as a form of hysteria depending upon religious excitement, and has its instances in every age and creed. Under its influence I have seen a feeble and foolish old woman rise, on the crowded floor of the great Mormon temple at Kirtland, Ohio, and speak with a force and eloquence of which she was entirely incompetent in her natural condition. It not unfrequently results in convulsions. The will is subject to a dominant idea. The mind can act with clearness upon that one subject; though on all others it is imbecile. Reason is useless with these patients; they see miracles where none exist; and are unable to call in the aid of memory to compare their present, with their past experience. It is a fortunate fact that this condition usually exhausts itself; that the bodily exertion and mental activity which it provokes, finally brings about a period of sluggishness and repose, from which renewed health may date. If this does not happen, the dominant idea becomes fixed, and a monomania of a confirmed insanity is the result.

The treatment in this disease, in any of its stages, is best found in the substitution of some other idea, and so removing the will from the chain which binds it.

5. *The Monomania for Deception.*—Although this article has been written with a freedom of expression not always prudent, and which I would not not be willing to use were I not now removed from all connection with those whose cases it describes, it is with a good deal of reluctance that I turn to this last feature of the mental symptom of hysteria. Few conditions of the human mind are so degrading as it; few can so excite the contempt and indifference of the physician; or the withdrawal of sympathy on the part of friends, as this strange manifestation.

Yet medical literature teems with cases of deceptions, cunningly conceived, and skilfully maintained; though to all appearance without motive or object to be gained. Prof. Hadley, of Buffalo Medical College, has in his possession a mass of common lime and sand mortar, which a lady professed to have passed from her bladder through the urethra. It is globular, and about three inches in diameter, weighing between ond and two pornds. A physician of intelligence in a neighboring county removed a calculus from the female urethra by forceps and published the case as illustrative of the facility with which large calculi might be removed without the necessity of the knife. Very much to his mortification the calculus proved to be a common brook pebble.

These deceptions, though somewhat laughable, are generally sources of great annoyance to friends; and, not unfrequently, of family disturbances and broils. A patient of my own insisted that she was covered by bruises received from her husband's cruelty. On an examination I could find no vestige of the pummeling she claimed to have received. She then said that the skin had resumed its natural color, though it was still extremely sore. She walked a mile to my office, through a wet snow, to get medicine rendered necessary by an abortion she said had happened to her on the previous night One evening she declared her intention to commit suicide, an announcement which her husband received very quietly. She locked herself up in her bedroom, and, after much groaning, was heard to open the window, thus conveying the idea that she had gone off to commit the fatal deed. An hour after her husband found her snugly stored away under the bed where she had been all the time.

It would be tiresome to recount all the numberless deceptions which this woman played off upon her family. The rationale of her case was simple. She had a slight uterine disease, manifested by hæmorrhage and other discharges. She had convinced herself that she was very sick, a fact which her husband unwisely refused to admit. With the dominant idea that she was ill and neglected strongly impressed upon her mind, and overruling all sense of decency, right, or truth, she was in fact a monomaniac. The necessary treatment could only be partially enforced. She was, however, measurably consoled by syringe, and a saturnine solution; together with the assurance that the family should be duly impressed with a sense of her alarming condition. The husband, however, (not a little mulish himself,) looked upon it as a case of peculiarly obstinate will, rather than any want of it. He yielded somewhat to the representation made, and a truce, but not a final peace, was established.

I believe that the longing for sympathy, or the conviction of neglect, lies at the bottom of all these deceptions. The lamentable results which sometimes follow from this condition, not unfrequently ending in insanity, render its treatment a matter of peculiar interest. It is safe to assume that any positive contradiction of the dominant idea will only result in further evil. The patient is not able to reason. She actually sees everything in a distorted light, and the only prudent course is to change in a gentle manner the course of her ideas. The substitution of new faces and new scenes, and the inculcation of the idea that all around her sympathize with her sufferings, will best remove the motive for deception, and restore the even tenor of the mind.

The selection of my cases from females mostly, might induce the idea that I consider the gentler sex as *alone* liable to this form of mental aberration.

I could easily correct this impression by details of cases occurring in strong muscular men. Among them was one, who, at every visit I made him uttered bitter complaints of neglect and abuse on the part of his family, and he sometimes entertained the idea that I was also his enemy, or rather that I would allow him to die, that I might reap the benefit of a post-mortem examination of his body. Both suppositions were equally preposterous. I cleared myself from his suspicion, by pretending great anxiety about my bill in case of his death. He subsequently procured a quantity of laudanum, and declared to me his intention of committing suicide. I saw at once that the suicidal impulse originated in a wish to secure the sympathy of those around him, and that he might bring grief and disgrace on his family. I ridiculed him for his purpose, and assured him that he would be laughed at, instead of pitied. He was at first very angry, and then very penitent.

A little more upon the peculiar causes of these conditions, and I shall close this long, and perhaps tedious, article.

Although both sexes are *liable* to hysterical delusions, yet it has always been a matter of note that females were more *frequently* the subject of them. A little examination of the causes which operate upon the different sexes will serve to explain this fact.

The generative system depends upon mental and moral causes for its activity. Like the secretion of tears, or the blush, it is entirely emotional in its causation. Necessarily it may react upon the moral senses. The mere fact that a blush has suffused the countenance, (however uncalled for.) is sufficient to produce a mental confusion which momentarily upsets the reasoning faculties, and renders its subject incompetent. So too, sexual irritations react upon the brain. The hypochrondria which attends involuntary seminal emissions is an instance of this reaction. Another is found in the common fact, that a person having gonorrhœa, is apt to be more alarmed and depressed in spirit, than if he were in a really dangerous condition from syphilis itself

The female has a larger extent of generative organism, of greater mobility, and subject to periodical activity. These circumstances, connected with her natural docility, and yielding or impressible character of will, render her peculiarly liable to the influence of dominant ideas; originating in those irritations, which, in either sex, are so apt to produce mental disturbances. The same facts, together with the presence of a spinal system predominating in activity over the brain, render her more subject to convulsive action. But where as often happens, the same relative conditions of will force, and spinal activity, obtain in the male, he is liable to conditions not differing in nervous, or mental character, from those of hysteria.

If, in this essay, I have wandered somewhat from the field of strictly medical inquiry into the difficult path of the relative influences of mind and matter, my apology must be found in the following quotations from Dr. Carpenter:

" In so far, then, as any part of the Nervous System merely reacts upon impressions which are made upon it we must regard its operations as *automatic;* and this as much when they give rise to Pschycical changes, as when they manifest themselves in evoking Muscular Movements, or in modifying the processes of Nutrition and Secretion.

" II. But the automatic actions of most parts of the Nervous System are subject, more or less completely, to the domination of the will, a power which is purely Pschycical, and of which we know nothing but what we learn from our own direct consciousness of its exercise. * * * * * * * * * * * * The functional disturbances of the Cerebrum induced by the irregular action of other parts of the Nervous System, is part of the Etiology of Insanity, which has been but very little attended to, but which deserves a careful study. Numerous examples of it are furnished by certain peculiar forms of disordered Mental action, which are connected with " hysterical " states of the female system, especially mutability and irritability of temper, and disposition to deceit; but we are probably also to refer to this cause, in part, at least, those very distressing states of mind which arise out of disorders in the sexual apparatus of the male, or even from irritation of neighboring parts."—Op. Cit.

